# Effect of *Oenothera odorata* Root Extract on Microgravity and Disuse-Induced Muscle Atrophy

**DOI:** 10.1155/2015/130513

**Published:** 2015-04-07

**Authors:** Yong-Hyeon Lee, Dong-Hyun Seo, Ji-Hyung Park, Kazuya Kabayama, Joerg Opitz, Kwang Ho Lee, Han-Sung Kim, Tack-Joong Kim

**Affiliations:** ^1^Division of Biological Science and Technology, Yonsei-Fraunhofer Medical Device Lab, College of Science and Technology, Yonsei University, Wonju 220-710, Republic of Korea; ^2^Department of Biomedical Engineering, Yonsei-Fraunhofer Medical Device Lab, College of Health Science, Yonsei University, Wonju 220-710, Republic of Korea; ^3^Department of Chemistry, Graduate School of Science, Osaka University, Osaka 5650871, Japan; ^4^Fraunhofer Institute for Ceramic Technologies and Systems IKTS Material Diagnostics, 01129 Dresden, Germany; ^5^Department of Biotechnology, College of Biomedical and Health Science, Research Institute of Inflammatory Diseases, Konkuk University, Chungju 380-701, Republic of Korea

## Abstract

Muscle atrophy, a reduction of muscle mass, strength, and volume, results from reduced muscle use and plays a key role in various muscular diseases. In the microgravity environment of space especially, muscle atrophy is induced by muscle inactivity. Exposure to microgravity induces muscle atrophy through several biological effects, including associations with reactive oxygen species (ROS). This study used 3D-clinostat to investigate muscle atrophy caused by oxidative stress *in vitro*, and sciatic denervation was used to investigate muscle atrophy *in vivo*. We assessed the effect of *Oenothera odorata* root extract (EVP) on muscle atrophy. EVP helped recover cell viability in C2C12 myoblasts exposed to microgravity for 24 h and delayed muscle atrophy in sciatic denervated mice. However, the expressions of HSP70, SOD1, and ceramide in microgravity-exposed C2C12 myoblasts and in sciatic denervated mice were either decreased or completely inhibited. These results suggested that EVP can be expected to have a positive effect on muscle atrophy by disuse and microgravity. In addition, EVP helped characterize the antioxidant function in muscle atrophy.

## 1. Introduction

Skeletal muscle atrophy can be induced by muscle disuse stemming from chronic inactivity (e.g., immobilization, bed rest, mechanical unloading, and spaceflight). These results can be loss of muscle mass, strength, and volume [1]. A recent study focused on the role of reactive oxygen species (ROS) and several complex biological effectors in causing and defining skeletal muscle atrophy [2]. ROS play an important regulatory role in skeletal muscle atrophy: during periods of muscle disuse ROS expression is increased by redox system disturbance [3]. Proteolysis involving the redox system evidently contributes to degradation of skeletal muscle protein during periods of disuse [4]. Space flight can increase free radical formation, thereby increasing the level of oxidative stress [5]. This effect is more pronounced in space flights of long duration [6]. An improvement in the balance of the antioxidant defense system will lessen the severity of oxidative stress induced by space flight [7]. Because muscle atrophy is caused by various factors, it is difficult to test it using the human. Hindlimb suspension and sciatic denervation are done with research animal models which can imitate a variety of conditions that induce human skeletal muscle atrophy [1], but it is still hard to simulate the space environment. Thus, the three-dimensional-clinostat (3D-clinostat) was developed to simulate microgravity for biological research [8, 9].

Several cell signals may regulate the oxidative stress associated with muscle atrophy. Heat shock proteins (HSPs), whose major function is to provide a molecular protein chaperone to regulate the assembly, rearrangement, and folding of proteins [10–13], have been known to protect cells against various stress-inducible apoptotic factors, including heat shock, tumor necrosis factor, starvation, and oxidative stress [14]. More importantly, HSP70 has been known as a promising molecule for regulation of the oxidative stress which induces apoptosis. A recent study has confirmed that HSP70 expression regulates oxidative stress [15]. Therefore, an increase in the expression of HSP70 can have a positive effect in muscle atrophy. Also, ceramides have been known as sphingolipid mediators in stress-inducible apoptosis [16]. Various stressor-factors leading to apoptosis have been reported to increase ceramide levels in several cell types, including myoblasts [17–22]. HSP70 may have antiapoptotic effects upstream of ceramide-induced caspases [23]. Additionally, Cu-Zn superoxide dismutase (SOD1), an antioxidant enzyme which catalyzes the dismutation of superoxide, is an important part of the antioxidant system for cells exposed to ROS. Absence of SOD1 leads to induced skeletal muscle atrophy by elevated oxidative stress [24].

In this study we confirmed that several cellular signal expression levels, including HSP70, ceramide, and SOD1 are affected by* Oenothera odorata* root extracts (EVP) in situations of oxidative stress induced by microgravity and disuse muscle atrophy. The oil of* O. odorata* seeds has previously been shown to have several beneficial effects on human health including antidiabetic, anti-inflammatory, and antipremenstrual activity [25, 26] but so far antioxidant effects of this plant have been unclear.

## 2. Materials and Methods

### 2.1. Materials

Penicillin-streptomycin was obtained from Lonza (Walkersville, MD, USA). An EZ-Cytox cell viability kit was purchased from Daeil Lab (Seoul, Korea). Antibody against HSP70 was purchased from Enzo Life Sciences, AG (Lausen, Switzerland). SOD1 and *β*-actin antibodies were purchased from Cell Signaling Technology (Danvers, MA, USA). N-acetyl-L-cysteine (NAC) and* ortho*-phthalaldehyde (OPA) were purchased from Sigma-Aldrich (St. Louis, MO, USA). Sphingomyelin, dihydrosphingomyelin, ceramide, and sphingolipid ceramide* N*-deacylase (SCDase) were purchased from Avanti Polar Lipids, Inc. (Alabaster, AL, USA). High Performance Thin Layer Chromatography (HPTLC) silica-gel plate, chloroform, and HPLC grade methanol were purchased from Merck (Darmstadt, Germany). Vectashield mounting medium with DAPI was purchased from Vector Laboratories (Burlingame, CA, USA). All other chemicals used were the highest analytical grade that is commercially available.

### 2.2. Preparation of* Oenothera odorata* Root Extract

The* O. odorata* plants were cultivated according to the good agricultural practices method of the Korea Rural Development Administration and were harvested during 2009 in Eumseong-gun, Korea (GPS: E 128°62′ N 36°56′). For sample preparation, the roots were extracted with ethanol three times at 25°C for three days. The extracts were filtrated through Whatman No. 1 filter paper (GE Healthcare, Buckinghamshire, UK) and combined followed by concentration using a rotary evaporator (EYELA N-1000, Japan) at 40°C. The obtained dried extracts were lyophilized and then powdered.

### 2.3. Cell Culture

C2C12 myoblasts were cultured in Dulbecco's Modified Eagle's Medium (DMEM; Sigma-Aldrich, St. Louis, MO, USA) supplemented with 10% (v/v) fetal bovine serum (FBS; Lonza, Walkersville, MD, USA), 100 *μ*g/mL penicillin-streptomycin, 8 mM N-(2-hydroxyelthyl) piperazine-N′-2-ethanesulfonic acid (HEPES), and 2 mM L-glutamine. C2C12 myoblasts were maintained at 37°C in a humidified 5% CO_2_ incubator.

### 2.4. 3D-Clinostat

A 3D-clinostat was used to simulate reduced gravity for gravity sensitive cell [27]. The 3D-clinostat was designed to fit the incubator. The size of the center plates was 160 mm by 106 mm. To operate the 3D-clinostat, two motors were used. The instrument could rotate linearly whose direction is clockwise (CW) and counter clockwise (CCW) and rotate at 2 rpm.

### 2.5. Animal Experiments

Male C57/BL6 mice (4-week-old) were purchased from Orient Bio (Gangneung, Korea) and were housed in wired cages with temperature 20–22°C and in 40–50% humidity. The Institutional Animal Care and Use Committee (IACUC, YWC-130204-1) at Yonsei University (Wonju, Korea) approved the protocol for this study. An attempt was made to minimize the pain of the animals. The sciatic nerve in the right leg of each mouse was surgically removed fragmentally to induce immobilization, and muscle atrophy occurred in the gastrocnemius muscle (GM) and soleus muscle (SM). These mice had induced muscle atrophy by sciatic denervation for 7 days. Then EVP (1-2 mg/kg) was injected by intramuscular injection 10 times over a 2-week period. The mice were sacrificed 21 days after sciatic denervation.

### 2.6. Microcomputed Tomography

Microcomputed tomography (micro-CT) images of the muscle in the tibia of each mouse (*n* = 5) were acquired 21 days after the induced muscle atrophy, using the micro-CT (SkyScan 1076, Bruker, Germany) at a resolution of 35 *μ*m, with the following parameters: 100 kV, 100 mA, 790 ms, and a rotation step of 1.2°. The mice were under anesthesia during the scanning. The beam-hardening errors were corrected to improve the quality of the micro-CT images by flat-field correction before scanning and beam-hardening correction during reconstruction. For the evaluation of muscle volume, 3D models of the tibia were reconstructed using CT-Analyzer 1.11 (CT-An 1.11, Bruker, Germany).

### 2.7. Cell Viability and Cytotoxicity

Cell viability was assessed with an EZ-Cytox cell viability kit following the manufacturer's instructions. Briefly, C2C12 myoblasts (2 × 10^4^ cells/well) were seeded into 96-well culture plates and incubated overnight in DMEM containing 10% (v/v) FBS at 37°C. When C2C12 myoblasts reached 70% confluence, the medium was replaced with serum-free DMEM containing various concentrations of EVP (0–50 *μ*g/mL) for 24 hr. To expose the microgravity, the medium was replaced with DMEM containing 10% (v/v) FBS. Microgravity was induced by 3D-clinostat for 24 hr. EZ-Cytox kit reagents were added to the medium, the C2C12 myoblasts were incubated for 1 hr, and then the optical density was determined at 450 nm using a microplate reader (BioTek Instruments Inc., Winooski, VT, USA).

### 2.8. Immunoblot Analysis

C2C12 myoblasts (2 × 10^5^ cells/well) were seeded into 6-well culture plates and incubated overnight in DMEM containing 10% (v/v) FBS at 37°C. Then, C2C12 myoblasts were cultured for 24 hr with or without EVP (50 *μ*g/mL) in serum-free DMEM. To expose the microgravity, the medium was replaced with DMEM containing 10% (v/v) FBS. Microgravity was induced by 3D-clinostat for 0–24 hr. Atrophy of GM and SM was induced by sciatic denervation with or without EVP (1-2 mg/kg) injection. Protein lysate was extracted using the PRO-PREP protein extraction kit (iNtRON, Sungnam-Si, Korea) following the manufacturer's instructions. The whole lysates were analyzed using sodium dodecyl sulfate-polyacrylamide gel electrophoresis (SDS-PAGE) on 10–15% polyacrylamide gels. The proteins were transferred to PVDF membrane (Bio-Rad, Hercules, CA, USA). The membranes were blocked overnight at 4°C in Tris-buffered saline containing 0.1% Tween-20 (TBS/T) and 5% skimmed milk powder and then incubated with each primary antibody. Blots were washed with TBS/T and incubated with each horseradish peroxidase-conjugated secondary antibody. Proteins were detected using an enhanced chemiluminescence (ECL) detection reagent for immunoblot analysis (GE Healthcare, Buckinghamshire, UK).

### 2.9. High Performance Liquid Chromatography (HPLC)

C2C12 myoblasts (2 × 10^5^ cells/well) were seeded into 6-well culture plates and incubated overnight in DMEM containing 10% (v/v) FBS at 37°C. Then, C2C12 myoblasts were cultured for 24 hr with or without EVP (50 *μ*g/mL) in serum-free DMEM. To expose the microgravity, the medium was replaced with DMEM containing 10% (v/v) FBS. Microgravity was induced by 3D-clinostat for 24 hr. Atrophy of GM and SM was induced by sciatic denervation with or without EVP (1-2 mg/kg) injection. C2C12 myoblasts and tissues from mice provided samples for lipid extraction. Ceramide quantification was performed according to previously described procedures [28] with some modifications. In brief, total lipids were extracted using chloroform/methanol/1 M NaCl solution (8 : 4 : 2.5, v/v/v) with an addition of dihydrosphingomyelin as an internal standard and dried. Then it was centrifuged at 13,000 rpm (4°C) for 10 min, and the supernatant was transferred to a clean 1.5-mL tube. The dried samples were dissolved in 30 *μ*L of chloroform/methanol (1 : 2, v/v) and spotted on an HPTLC silica-gel plate for ceramide separation. Then ceramide was converted simultaneously to sphingosine by ceramidase in a reaction buffer (pH 7.5) containing 25 mM Tris-HCl buffer, 1% sodium chlorate, and 15% fatty acid-free bovine serum albumin. The released sphingosine from ceramide was analyzed using HPLC following* ortho*-phthalaldehyde derivatization.

### 2.10. Immunocytochemistry

C2C12 myoblasts (2 × 10^5^ cells/well) were seeded on a cover glass into 6-well culture plates and incubated overnight in DMEM containing 10% (v/v) FBS at 37°C. Then, C2C12 myoblasts were cultured for 24 hr with or without EVP (50 *μ*g/mL) in serum-free DMEM. To expose the microgravity, the medium was replaced with DMEM containing 10% (v/v) FBS. Microgravity was induced by 3D-clinostat for 24 hr. C2C12 myoblasts were fixed in 4%* para*-formaldehyde, 0.1% Triton-X treated for 25 min, and 3% BSA blocking for 30 min in shaking rocker. C2C12 myoblasts were incubated with antibodies against SOD1 (1 : 1000 dilution) overnight at 4°C. C2C12 myoblasts were washed three times with PBS and incubated with secondary antibodies, Alexa Fluor 488 anti-rabbit IgG (Invitrogen, Seoul, Korea), for 3 hr at 4°C. After being washed with PBS, C2C12 myoblasts were stained and mounted with DAPI-mounting media. Images were acquired with an LSM710 confocal microscope (Zeiss, Jena, Germany). During confocal microscopic observation, all the images were taken using the same settings.

### 2.11. Statistical Analysis

Experimental results are expressed as the mean ± SD. One-way analysis of variance (ANOVA) was followed by Tukey's multiple comparison test using GraphPad Prism 5.0 software (GraphPad Software, Inc., San Diego, CA, USA). Values of *P* < 0.01 were considered to indicate statistically significant differences.

## 3. Results

### 3.1. Effect of EVP on Microgravity by 3D-Clinostat in C2C12 Myoblast

**Figure 1:**
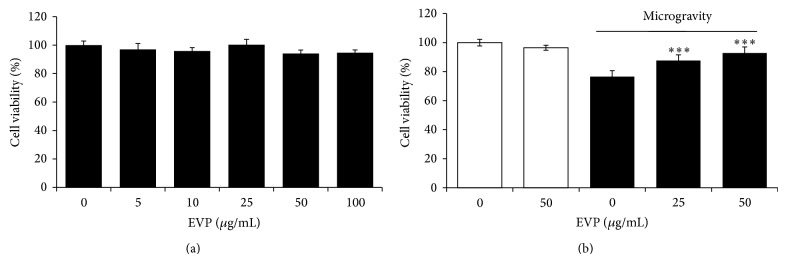
Effect of EVP on microgravity by 3D-Clinostat in C2C12 myoblasts. (a) Cytotoxicity of EVP in C2C12 myoblasts. C2C12 myoblasts were cultured in 96-well plates until confluent, and the medium was replaced with serum-free medium with or without EVP (0–100 *μ*g/mL) for 24 hr. The EZ-Cytox reagent was added to the medium, and C2C12 myoblasts were incubated for 1 hr. The optical density was determined at 450 nm using a microplate reader. Shown are the mean values (±SD) from three experiments. (b) Cell viability on microgravity by 3D-clinostat. C2C12 myoblasts were cultured in 96-well plates until confluent and the medium then replaced a serum-free medium with the EVP (0–50 *μ*g/mL). After preincubating for 24 hr, 3D-clinorotation was subjected for 24 hr with DMEM containing 10% (v/v) FBS. After PBS washing, the EZ-Cytox reagent was added to the medium, and the C2C12 myoblasts were incubated for an additional 1 hr. The optical density was determined at 450 nm by using a microplate reader. The cell viability was calculated by using the following equation: cell viability (%) = [(absorbance of the 3D-clinorotation sample/absorbance of the 3D-unrotated control) × 100]. Each value represents the mean (±SD) from three experiments, each performed in triplicate. ^∗∗∗^ *P* < 0.0001 versus microgravity alone.

The microgravity environment of space results in muscle atrophy due to muscle disuse and inactivity. We hypothesized that EVP can have an antioxidant effect on microgravity-induced oxidative stress in C2C12 myoblasts. EVP showed no evidence of cytotoxicity within the dose range used in C2C12 myoblasts (Figure 1(a)). Microgravity (24 hr) reduced cell viability by approximately 73.31%. However, EVP (0–50 *μ*g/mL) recovered cell viability significantly in a concentration-dependent manner (Figure 1(b)).

### 3.2. Effect of EVP on HSP70 Expression by Microgravity in C2C12 Myoblasts

**Figure 2:**
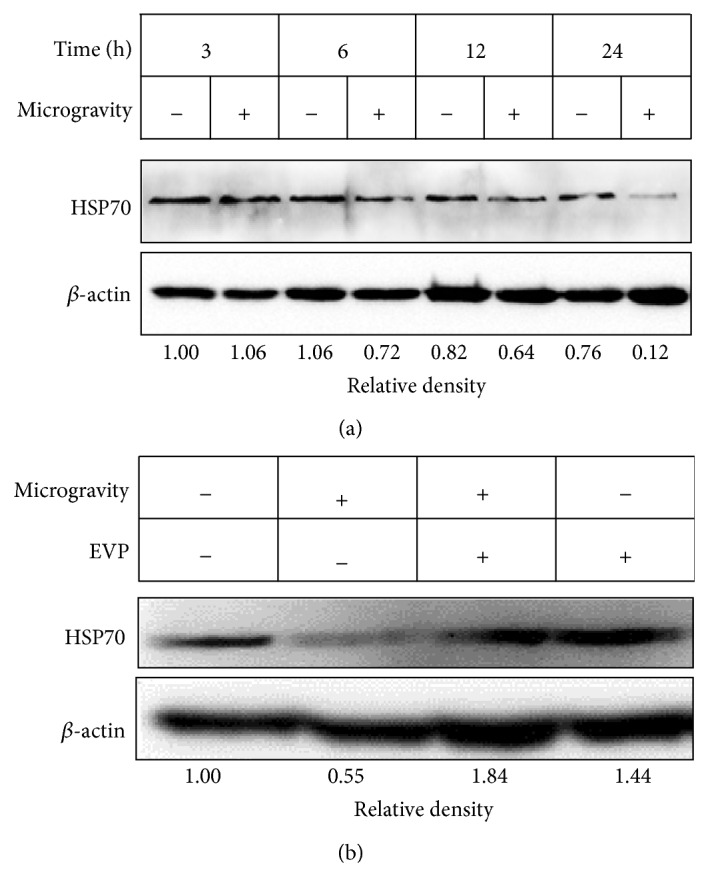
Effect of EVP on HSP70 expression by microgravity in C2C12 myoblasts. (a) Effect of microgravity on HSP70 expression. After culturing with media for 24 hr, myoblasts were subjected to 3D-clinorotation for 0–24 hr. HSP70 and *β*-actin were analyzed by immunoblot analysis using specific antibodies. (b) Effect of EVP on microgravity by 3D-clinostat. C2C12 myoblasts were cultured in 12-well plates until confluent, and the medium was then replaced to serum-free medium with or without the EVP (50 *μ*g/mL) for 24 hr. 3D-clinorotation subjected for 24 hr with DMEM containing 10% (v/v) FBS. Protein lysate was extracted using the PRO-PREP Protein extraction Kit. The whole lysates were analyzed using sodium dodecyl sulfate-polyacrylamide gel electrophoresis (SDS-PAGE) on 12% polyacrylamide gels. HSP70 and *β*-actin were analyzed by immunoblot analysis using specific antibodies. Immunoblot was analyzed by densitometry and the inserts display representative blots of four similar independent experiments, respectively.

The protective effect of HSP70 is related to the inhibition of apoptosis. Several studies have shown that HSP70 protects cells from stress induced cell death [26, 27]. We recorded the time course data of HSP70 expression patterns under a microgravity condition induced by 3D-clinostat (Figure 2(a)). Exposure to microgravity for 24 hr significantly decreased HSP70 compared to the other time groups. In addition, the expression of HSP70 increased in the group treated with EVP (50 *μ*g/mL) (Figure 2(b)). Thus, we confirmed the relationship of microgravity and HSP70 expression. It is likely that HSP70 played a role in preventing the microgravity-induced cellular stress of C2C12 myoblasts.

### 3.3. Effect of EVP on Oxidative Stress by Microgravity in C2C12 Myoblasts

**Figure 3:**
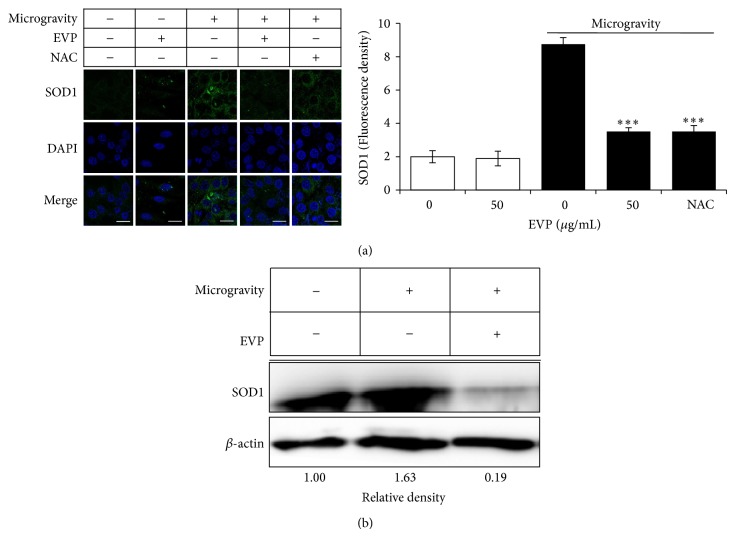
Effect of EVP on oxidative stress by microgravity in C2C12 myoblasts. (a) Effect of EVP on SOD1 expression using immunocytochemistry. Representative images (up) and quantitative analysis (down) of fluorescence density of SOD1 in C2C12 myoblasts. C2C12 myoblasts were cultured in 35 mm cover-glass-bottom dish, and the medium was then replaced to serum-free medium with or without NAC (2 mM) or EVP (50 *μ*g/mL). After 24 hr, 3D-clinorotation was subjected for 24 hr with DMEM containing 10% (v/v) FBS. Images were acquired with a LSM710 confocal microscope after immunofluorescence staining with SOD1 and Alexa 488 antibodies (green). C2C12 myoblasts were stained with DAPI to visualize nuclei (blue). Images are shown the mean values (±SD) from three experiments. Scale bar: 20 *μ*m. NAC: N-acetyl cysteine. ^∗∗∗^ *P* < 0.0001 versus microgravity alone. (b) Effect of EVP on SOD1 expression using immunoblot analysis. C2C12 myoblasts were cultured in 12-well plates until confluent, and the medium was then replaced to serum-free medium with or without the EVP (50 *μ*g/mL) for 24 hr. 3D-clinorotation was subjected for 24 hr with DMEM containing 10% (v/v) FBS. Protein lysate was extracted using the PRO-PREP Protein extraction Kit. The whole lysates were analyzed using sodium dodecyl sulfate-polyacrylamide gel electrophoresis (SDS-PAGE) on 12% polyacrylamide gels. SOD1 and *β*-actin were analyzed by immunoblot analysis using specific antibodies. Immunoblot was analyzed by densitometry and the inserts display representative blots of four similar independent experiments, respectively.

In this study, we used a 3D-clinostat to investigate the protective effect of EVP against microgravity-induced oxidative stress. Microgravity was induced by 3D-clinostat for 24 hr with or without EVP (50 *μ*g/mL). SOD1, activated by cellular ROS, is antioxidant enzyme located in the cytoplasm. Using immunocytochemistry, we observed a significant increase on SOD1 expression level upon microgravity-induced oxidative stress. However, EVP (50 *μ*g/mL) suppressed SOD1 expression level, when compared to the untreated group (Figure 3(a)). Also, we used immunoblot analysis to assess cellular protein levels. Expression of SOD1 increased significantly on oxidative stress by microgravity; however, EVP (50 *μ*g/mL) downregulated protein expression levels (Figure 3(b)).

### 3.4. Effect of EVP on Ceramide Level by Microgravity in C2C12 Myoblasts

**Figure 4:**
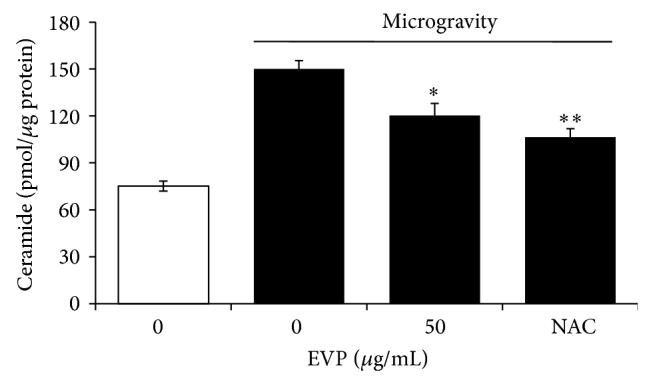
Effect of EVP on ceramide level by microgravity in C2C12 myoblasts. After culturing with media for 24 hr, NAC (2 mM) or EVP (50 *μ*g/mL) was added with serum-free medium for 24 hr. Then 3D-clinorotation was subjected for 24 hr with DMEM containing 10% (v/v) FBS. Lipids were extracted from the C2C12 myoblasts pellets with ethanol containing C_17_-ceramide as internal standard. The C_17_- and C_18_-ceramides in the extract were separated by thin-layer chromatography and deacylated with ceramidase. Ceramide quantification was performed by HPLC, as described in Section 2. Each value represents the mean (±SD) from three experiments. NAC:* N*-acetyl cysteine. ^∗^ *P* < 0.01 and ^∗∗^ *P* < 0.001 versus microgravity alone.

Recent several studies have suggested that induction of ceramide is caused by various stressors [29, 30]. HPLC was used to quantify ceramide upon microgravity by 3D-clinosstat in C2C12 myoblasts. Microgravity was induced by 3D-clinostat for 24 hr with or without EVP (50 *μ*g/mL). Exposure to microgravity for 24 hr caused the 2.01-fold increase of endogenous ceramide levels. However, EVP (50 *μ*g/mL) decreased endogenous ceramide levels by 23.61% (Figure 4).

### 3.5. Effects of EVP on Disuse Muscle Atrophy by Sciatic Denervation in Mice

**Figure 5:**
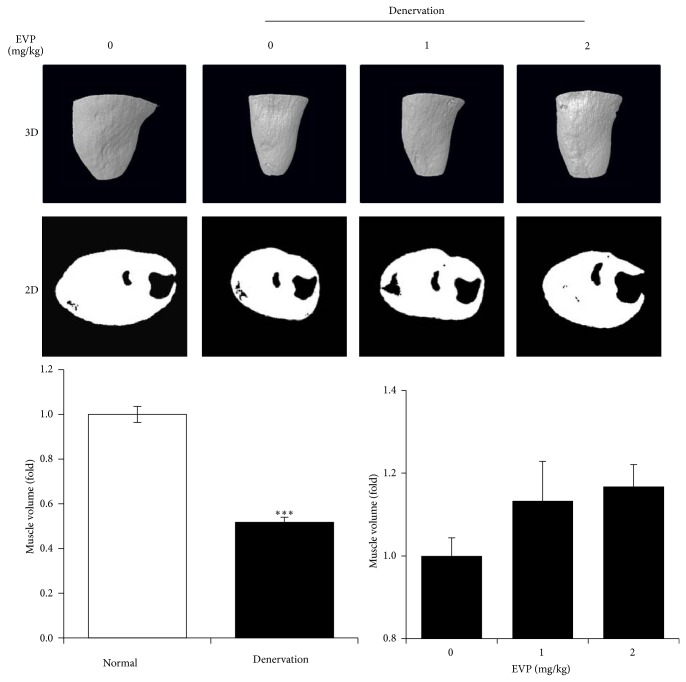
Effects of EVP on disuse muscle atrophy by sciatic denervation in mice. Measurement to GM and SM of sciatic denervated mice using micro-CT. Images of GM and SM in sciatic denervated mice (*n* = 5) were acquired at 21 days after the induced muscle atrophy. For evaluation of GM and SM volume, three-dimensional (3D) and two-dimensional (2D) reconstructed images were analyzed using CT-Analyzer 1.11 in control, sciatic denervated, and EVP groups. Each value represents the mean (±SD) from five mice. ^∗∗∗^ *P* < 0.0001 versus sciatic denervation alone.

Muscle atrophy induced by sciatic denervation was investigated with micro-CT in mice. Artificial reduction of muscular activity via sciatic denervation caused muscle atrophy. We investigated muscle volume and other factors considering the distribution of muscle. GM and SM volumes of sciatic denervated mice were lower than control mice. However, muscle volume of the EVP group was higher than the non-EVP-treated group of sciatic denervated mice (Figure 5).

### 3.6. Effect of EVP on HSP70 Expression by Disuse Muscle Atrophy in Mice

**Figure 6:**
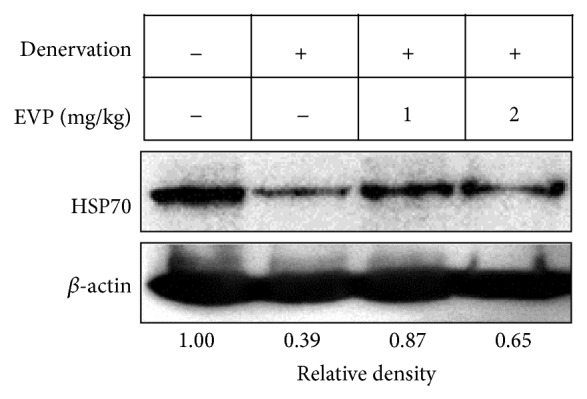
Effect of EVP on HSP70 expression by disuse muscle atrophy in mice. GM of sciatic denervated mice were compared with control and EVP groups. Protein lysate was extracted using the PRO-PREP Protein extraction Kit. The whole lysates were analyzed using sodium dodecyl sulfate-polyacrylamide gel electrophoresis (SDS-PAGE) on 12% polyacrylamide gels. HSP70 and *β*-actin were analyzed by immunoblot analysis using specific antibodies. Immunoblot was analyzed by densitometry and the inserts display representative blots of four similar independent experiments, respectively.

In this study, we investigated how muscle volume loss during disuse atrophy correlates with HSP70 expression using sciatic denervated mice. HSP70 was downregulated in denervated mice. However, HSP70 expression levels were higher in the EVP group than the non-EVP-treated group of sciatic denervated mice (Figure 6). This result suggests that EVP prevents the muscle atrophy caused by sciatic denervation in mice.

### 3.7. Effect of EVP on Ceramide Level by Disuse Muscle Atrophy in Mice

**Figure 7:**
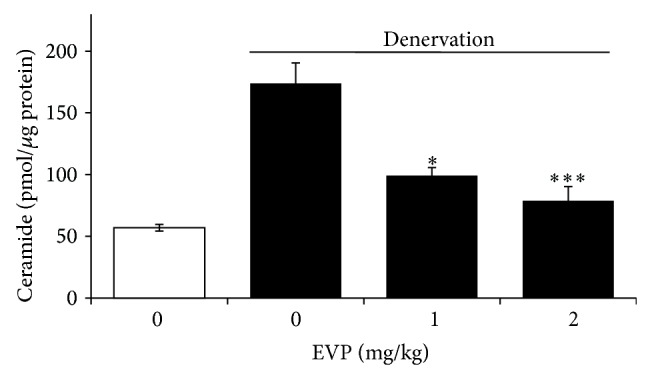
Effect of EVP on ceramide level by disuse muscle atrophy in mice. GM of sciatic denervated mice were compared with control and EVP groups. Lipids were extracted from whole lysate of GM and SM with ethanol containing C_17_-ceramide as internal standard. The C_17_- and C_18_-ceramides in the extract were separated by thin-layer chromatography and deacylated with ceramidase. The C_17_ and C_18_ sphingosine were derived with OPA fluorescence dye. Ceramide quantification was performed by HPLC. Each value represents the mean (±SD) from five mice. ^∗^ *P* < 0.01 and ^∗∗∗^ *P* < 0.0001 versus sciatic denervation alone.

We confirmed the relation of ceramide in sciatic denervation induced muscle atrophy. According to another study, it is widely known that the antiapoptotic function of HSP70 is repressed by ceramide as a proapoptotic factor [17]. We found that the amount of ceramide increased in sciatic denervated mice. The ceramide level exhibited an approximately 3.01-fold increase in the GM and SM of sciatic denervated mice. However, the ceramide level was reduced 17.32% and 38.16% in the EVP group (1-2 mg/kg injection) compared to the non-EVP-treated group of sciatic denervated mice (Figure 7).

## 4. Discussion

This study has the objective to identify effects of new natural products in muscle atrophy. It is absolutely necessary for projects with long-term periods in space and for space-related technology. Our findings can also be applied to skeletal muscular diseases such as muscle atrophy, which is closely related to oxidative stress [31–33]. Furthermore, because normal muscle cells and myoblasts are sensitive to oxidative stress, promoting antioxidative protection effects is a useful strategy to prevent oxidative injury or to delay the progress of related diseases [1].

HSPs regulate the muscular system as intracellular chaperones. HSP70, especially, has a protective effect against various stresses [34–38]. In this study, HSP70 significantly decreased oxidative stress* in vitro* and* in vivo*. Additionally, HSP70 increased with exposure to EVP. Ceramide as a proapoptotic factor was increased by microgravity (*in vitro*) and sciatic denervation (*in vivo*). However, EVP reduced the amount of endogenous ceramide. Thus, the protective effects of HSP70 are closely linked to apoptosis inhibition.

Recently, several studies suggest that ROS in skeletal muscle contribute to disuse muscle atrophy [39, 40]. In this study, several stressors (intracellular ROS, microgravity, and sciatic denervation) increased SOD1; However, EVP decreased the expression of SOD1 upon oxidative stress* in vitro* and* in vivo*. One study suggested that intramuscular injection of the flavonoid quercetin into the GM effectively prevented loss of muscle weight in hindlimb suspension mice [41]. In another study, rats treated with the lipid-soluble antioxidant, vitamin E, showed disuse muscle atrophy improvement of approximately 20% [39, 40].

Microgravity and sciatic denervation both caused skeletal muscle atrophy. EVP showed an antioxidant capacity that can prevent muscle atrophy from various stresses including microgravity, ROS, and oxidative stress. Systematic studies of biological effects and changes in muscle atrophy via microgravity and disuse have not been completed. Our study suggests a new effect mechanism and a method for treating the muscle atrophy caused by microgravity-induced oxidative stress. Muscle atrophy is also an issue in aging societies because it is a common problem for bed-ridden patients [42]. Using natural products, we aim to find new therapeutic agents for muscle atrophy, which can contribute to improved space technology competitiveness as well as medical advances.
